# Supplementary material for: Postmastectomy radiotherapy in pN1 breast cancer: Survival outcomes and prognostic factors from a single-institution cohort
Source: PLoS One. 2026 Jun 1;21(6):e0341754. doi: 10.1371/journal.pone.0341754 (PMC13225653; doi:10.1371/journal.pone.0341754)
Supplement: S1 Table — HR: Hazard Ratio assessing risk of RFS events for 1-unit increase, or for specific category vs reference; CI: Confidence Interval; P: p-value testing HR = 1 from Wald Test. N/A: not applicable due to the small number of events in a group. Note: For a variable with missing value, we clarified the evaluable number of participants and events after excluding the missing values. (DOCX) [file pone.0341754.s001.docx]

| **Variable** | **Category** | **HR (95%CI)** | **P** |
| --- | --- | --- | --- |
| ***Subset univariable Analyses: 1. Triple Negative Receptor (n=9, 5 events)*** | | | |
| Age | 1-unit increase | 1.02 (0.96, 1.08) | 0.758 |
|  | >60 vs ≤60 (ref) | Not Estimable | 0.998 |
| Race | White vs Black (ref) | Not Estimable | 0.997 |
| Ethnicity | Hispanic vs Non-Hispanic (ref) | 0.61 (0.10, 3.79) | 0.599 |
| Laterality | Left vs Right (ref) | 0.75 (0.10, 5.44) | 0.773 |
| Cancer Type  (n=7, 3 events) | N/A | N/A | N/A |
| Chemotherapy | N/A | N/A | N/A |
| Multiple Chemotherapy | Multiple Agents vs  None/NOS/Single Agent (ref) | 1.61 (0.17, 15.53) | 0.681 |
| Hormone Therapy | N/A | N/A | N/A |
| Immunotherapy | Yes vs No (ref) | 2.85 (0.25, 32.09) | 0.397 |
| Tumor Size | p2 vs p1Mi/p1a/p1b/p1c (ref) | 0.18 (0.02, 2.05) | 0.168 |
| Surgical Pathological Stage | 2a/2b vs 1a/1b/1c (ref) | 0.18 (0.02, 2.05) | 0.168 |
| PMRT | Yes vs No (ref) | 1.38 (0.14, 14.14) | 0.785 |
| Number of Positive Lymph Nodes | 2-3 vs 1 (ref) | 2.85 (0.25, 32.09) | 0.397 |
| ***2. Non-Triple Negative Receptor (n=45, 24 events)*** | | | |
| Age | 1-unit increase | 1.04 (1.00, 1.09) | 0.061 |
|  | >60 vs ≤60 (ref) | 1.41 (0.61, 3.26) | 0.422 |
| Race | White vs Black (ref) | 1.60 (0.37, 6.90) | 0.528 |
| Ethnicity | Hispanic vs Non-Hispanic (ref) | 0.95 (0.39, 2.32) | 0.913 |
| Laterality  (n=44, 23 events) | Left vs Right (ref) | 0.57 (0.25, 1.31) | 0.186 |
| Cancer Type  (n=40, 21 events) | ILC vs IDC (ref) | 0.84 (0.19, 3.65) | 0.816 |
| Chemotherapy  (n=43, 23 events) | NOS/Single/Multiple vs  None (ref) | 0.54 (0.22, 1.31) | 0.172 |
| Multiple Chemotherapy (n=43, 23 events) | Multiple Agents vs  None/NOS/Single Agent (ref) | 0.64 (0.28, 1.45) | 0.282 |
| Hormone Therapy  (n=44, 23 events) | Yes vs No (ref) | 0.21 (0.08, 0.57) | **0.002** |
| Immunotherapy | Yes vs No (ref) | 1.61 (0.37, 6.98) | 0.525 |
| Tumor Size | p2 vs p1Mi/p1a/p1b/p1c (ref) | 1.54 (0.65, 3.66) | 0.323 |
| Surgical Pathological Stage | 2a/2b vs 1a/1b/1c (ref) | 1.90 (0.82, 4.40) | 0.135 |
| PMRT | Yes vs No (ref) | 0.50 (0.20, 1.21) | 0.124 |
| Number of Positive Lymph Nodes (n= 40, 22 events) | 2-3 vs 1 (ref) | 2.53 (1.07, 5.96) | **0.034** |
