# Supplementary material for: Postmastectomy radiotherapy in pN1 breast cancer: Survival outcomes and prognostic factors from a single-institution cohort
Source: PLoS One. 2026 Jun 1;21(6):e0341754. doi: 10.1371/journal.pone.0341754 (PMC13225653; doi:10.1371/journal.pone.0341754)
Supplement: S2 Table — HR: Hazard Ratio assessing risk of death for 1-unit increase, or for specific category vs reference; CI: Confidence Interval; P: p-value testing HR = 1 from Wald Test. N/A: not applicable due to the small number of events in a group. Note: For a variable with missing value, we clarified the evaluable number of participants and events after excluding the missing values. (DOCX) [file pone.0341754.s002.docx]

| **Variable** | **Category** | **HR (95%CI)** | **P** |
| --- | --- | --- | --- |
| ***Subset univariable Analyses: 1. Triple Negative Receptor (n=9, 5 events)*** | | | |
| Age | 1-unit increase | 1.02 (0.96, 1.08) | 0.625 |
|  | >60 vs ≤60 (ref) | 3.24 (0.20, 51.9) | 0.406 |
| Race | White vs Black (ref) | 0.31 (0.02, 4.94) | 0.406 |
| Ethnicity | Hispanic vs Non-Hispanic (ref) | 0.29 (0.03, 2.66) | 0.276 |
| Laterality | Left vs Right (ref) | Not Estimable | 0.997 |
| Cancer Type  (n=7, 3 events) | N/A | N/A | N/A |
| Chemotherapy | N/A | N/A | N/A |
| Multiple Chemotherapy | Multiple Agents vs  None/NOS/Single Agent (ref) | 0.66 (0.11, 4.16) | 0.662 |
| Hormone Therapy | N/A | N/A | N/A |
| Immunotherapy | Yes vs No (ref) | 1.31 (0.13, 13.55) | 0.819 |
| Tumor Size | p2 vs p1Mi/p1a/p1b/p1c (ref) | 0.41 (0.06, 2.93) | 0.373 |
| Surgical Pathological Stage | 2a/2b vs 1a/1b/1c (ref) | 0.41 (0.06, 2.93) | 0.373 |
| PMRT | Yes vs No (ref) | 2.68 (0.23, 31.22) | 0.431 |
| Number of Positive Lymph Nodes | 2-3 vs 1 (ref) | 1.31 (0.13, 13.55) | 0.819 |
| Recurrence Occurred | Yes vs No (ref) | 2.82 (0.31, 25.95) | 0.359 |
| Recurrence Type | Local/Nodal vs  No Recurrence (ref) | 2.63 (0.14, 47.91) | 0.514 |
|  | Systemic vs  No Recurrence (ref) | 2.88 (0.30, 28.07) | 0.362 |
| ***2. Non-Triple Negative Receptor (n=45, 7 events)*** | | | |
| Age | 1-unit increase | 1.14 (1.01, 1.29) | **0.039** |
|  | >60 vs ≤60 (ref) | 2.59 (0.58, 11.65) | 0.214 |
| Race | White vs Black (ref) | Not Estimable | 0.996 |
| Ethnicity | Hispanic vs Non-Hispanic (ref) | 1.76 (0.39, 7.88) | 0.459 |
| Laterality  (n=44, 7 events) | Left vs Right (ref) | 0.67 (0.15, 3.07) | 0.610 |
| Cancer Type  (n=40, 6 events) | ILC vs IDC (ref) | 1.47 (0.17, 12.64) | 0.725 |
| Chemotherapy  (n=43, 7 events) | NOS/Single/Multiple vs  None (ref) | 0.33 (0.07, 1.50) | 0.152 |
| Multiple Chemotherapy (n=43, 7 events) | Multiple Agents vs  None/NOS/Single Agent (ref) | 0.73 (0.16, 3.29) | 0.686 |
| Hormone Therapy  (n=44, 7 events) | Yes vs No (ref) | 0.12 (0.03, 0.56) | **0.007** |
| Immunotherapy | Yes vs No (ref) | Not Estimable | 0.996 |
| Tumor Size | p2 vs p1Mi/p1a/p1b/p1c (ref) | 2.34 (0.45, 12.04) | 0.311 |
| Surgical Pathological Stage | 2a/2b vs 1a/1b/1c (ref) | 2.87 (0.56, 14.79) | 0.208 |
| PMRT | Yes vs No (ref) | 0.30 (0.04, 2.46) | 0.260 |
| Number of Positive Lymph Nodes (n= 40, 7 events) | 2-3 vs 1 (ref) | 3.21 (0.71, 14.56) | 0.130 |
| Recurrence Occurred | Yes vs No (ref) | 2.45 (0.29, 20.40) | 0.230 |
| Recurrence Type | Local/Nodal vs  No Recurrence (ref) | 4.36 (0.39, 48.18) | 0.175 |
|  | Systemic vs  No Recurrence (ref) | 2.01 (0.22, 18.02) | 0.533 |
| HR: Hazard Ratio assessing risk of death for 1-unit increase, or for specific category vs reference; CI: Confidence Interval; P: p-value testing HR =1 from Wald Test.  N/A: not applicable due to the small number of events in a group.  **Note:** For a variable with missing value, we clarified the evaluable number of participants and events after excluding the missing values. | | | |
