# Supplementary material for: Postmastectomy radiotherapy in pN1 breast cancer: Survival outcomes and prognostic factors from a single-institution cohort
Source: PLoS One. 2026 Jun 1;21(6):e0341754. doi: 10.1371/journal.pone.0341754 (PMC13225653; doi:10.1371/journal.pone.0341754)
Supplement: Attachment — Submitted filename: report_10.64898+2026.02.13.26346274.pdf [file pone.0341754.s003.pdf]

ScreenIT Report

This is an automated report for 10.64898+2026.02.13.26346274.pdf. This report was solicited by the PLOS One editorial team and provided by ScreenIT.

ScreenIT is an independent group of scientists developing automated tools that analyze academic papers. A set of automated tools screened your submitted manuscript and provided the report below. Each tool was created by your academic colleagues with the goal of helping authors.

The tools look for factors that are important for transparency, rigor and reproducibility, and we hope that the report might help you to improve reporting in your manuscript. Within the report you will find links to more information about the items that the tools check. These links include helpful papers, websites, or videos that explain why the item is important.

While our screening tools aim to improve and maintain quality standards they may, on occasion, miss nuances specific to your study type or flag something incorrectly. Each tool has limitations that are described on the ScreenIT website. The tools screen the main file for the paper; they are not able to screen supplements stored in separate files.

Please note that the Academic Editor had access to these comments while making a decision on your manuscript. The Academic Editor may ask that issues flagged in this report be addressed.

If you would like to provide feedback on the ScreenIT tool, please email the team at ScreenIt@bih-charite.de. If you have questions or concerns about the review process, please contact the PLOS One office at plosone@plos.org.

How to read the report:

- no action required
- review for potential issues
- a critical issue was found by a tool
- may not be applicable to your paper

- more information about this item can be obtained using this link

Rigor

n/a	Ethics	not required.
✓	Inclusion and Exclusion Criteria	"Inclusion criteria Inclusion criteria were as follows: age between 5 and 17 years at the time of evaluation; confirmed sickle cell disease based on hemoglobin electrophoresis or molecular testing (genotypes SS, SC, S, or S); confirmed asthma, defined by: o either evidence of reversible airflow obstruction on spirometry (increase in FEV 12% after bronchodilator administration), o or a physician-diagnosed asthma established by a pediatrician and documented in the medical record, reflecting real-life clinical practice."
✓	Flow charts and attrition	Thank you for including a study flow chart and information about attrition and exclusions to help readers evaluate the risk of bias.<br><br>Sentence about attrition: "not required."
✓	Sex as a biological variable	"Statistical significance was defined as a p value < 0.05. 4 Results Among children with sickle cell disease and confirmed asthma included in the analysis (n = 138), the median age was 8.0 years (IQR: 6.011.0), and 45.7% were male."
✓	Subject Demographics	Age: "Inclusion criteria Inclusion criteria were as follows: age between 5 and 17 years at the time of evaluation; confirmed sickle cell disease based on hemoglobin electrophoresis or molecular testing (genotypes SS, SC, S, or S); confirmed asthma, defined by: o either evidence of reversible airflow obstruction on spirometry (increase in FEV 12% after bronchodilator administration), o or a physician-diagnosed asthma established by a pediatrician and documented in the medical record, reflecting real-life clinical practice."
?	Randomization	Not detected. If you performed an experimental study, please specify whether participants, animals or samples were randomly

		assigned to treatments or groups, and specify the randomization procedure.
?	Blinding	Not detected. Please specify whether blinding was used at various phases of the experiment (e.g., blinding of patients, caregivers, outcome assessments, data analysis).
?	Power Analysis	Not detected. Please state how you determined the sample size for the study. If you performed a power or sample size calculation, please provide details.
n/a	Replication	not required.

Generated by Barzooka (flow charts), SciScore (everything else).
SciScore only screens the text of the methods section; we apologize if the tool has missed items reported elsewhere in the paper or supplements.

Resources

No key resources detected.
Generated by SciScore. SciScore only screens the text of the methods section; we apologize if the tool has missed items reported elsewhere in the paper or supplements.

Transparency

?	Open code	If you wrote code to analyze your data, please consider sharing the code in a public repository. This makes it easier for others to reproduce your analyses, and may also aid others seeking to analyze similar datasets.
?	Open data	If permitted, sharing data on a public repository with a persistent identifier (a DOI or accession number) can improve reproducibility and make it easier for other scientists to expand on your work. Papers with open data are cited more often than papers without open data. Some institutions have an expert who can provide advice on data sharing.
✓	Self-acknowledged limitations	"Strengths and limitations Limitations include the observational design of the study and the sample size, which may have limited statistical power to detect independent associations, particularly for parasitological factors. These findings should therefore be

		interpreted as hypothesisgenerating and warrant confirmation in larger cohorts."
✓	Conflict of interest statement	ⓘ Thank you for stating any conflicts of interest.
✓	Funding statement	ⓘ Thank you for reporting your funding source(s), or stating that there was no specific funding for this work.
n/a	Registration statement	ⓘ A registered protocol makes the planned research project more transparent and can protect against bias. Please consider registering future confirmatory studies.

Generated by ODDPub, limitation-recognizer, rtransparent, trial-identifier.

Figures

✓	No bar graphs of continuous data	ⓘ Thank you for not using bar graphs to present continuous data. Many different datasets and data distributions can lead to the same bar graph, and the actual data may suggest different conclusions from the summary statistics alone.
✓	No rainbow color maps	ⓘ Thank you for not using rainbow colormaps. Rainbow color maps distort readers' perception of the data by introducing visual artifacts and are not accessible to colorblind readers.

Generated by JetFighter, Barzooka.
